# Temporally extended goal recognition in fully observable non-deterministic domain models: Temporally extended goal recognition in FOND planning

**DOI:** 10.1007/s10489-023-05087-1

**Published:** 2023-12-14

**Authors:** Ramon Fraga Pereira, Francesco Fuggitti, Felipe Meneguzzi, Giuseppe De Giacomo

**Affiliations:** 1 University of Manchester, Manchester, UK; 2 IBM Research AI, Cambridge, USA; 3 Sapienza University, Rome, Italy; 4 York University, Toronto, Canada; 5 University of Aberdeen, Scotland, UK; 6 University of Oxford, England, UK

**Keywords:** Automated planning, Goal recognition, Non-deterministic planning, Linear temporal logic.

## Abstract

*Goal Recognition* is the task of discerning the intended goal that an agent aims to achieve, given a set of goal hypotheses, a domain model, and a sequence of observations (i.e., a sample of the plan executed in the environment). Existing approaches assume that goal hypotheses comprise a single conjunctive formula over a single final state and that the environment dynamics are deterministic, preventing the recognition of temporally extended goals in more complex settings. In this paper, we expand goal recognition to *temporally extended goals* in *Fully Observable Non-Deterministic* (fond) planning domain models, focusing on goals on finite traces expressed in *Linear Temporal Logic* (ltl$_f$) and *Pure-Past Linear Temporal Logic* (ppltl). We develop the first approach capable of recognizing goals in such settings and evaluate it using different ltl$_f$ and ppltl goals over six fond planning domain models. Empirical results show that our approach is accurate in recognizing temporally extended goals in different recognition settings.

## Introduction

*Goal Recognition* is the task of recognizing the intentions of autonomous agents or humans by observing their interactions in an environment. Existing work on goal and plan recognition addresses this task over several different types of domain settings, such as plan-libraries [4], plan tree grammars [19], classical planning domain models [31, 34, 35, 37], stochastic environments [36], continuous domain models [22], incomplete discrete domain models [29], and approximate control models [30]. Despite the ample literature and recent advances, most existing approaches to *Goal Recognition as Planning* cannot recognize *temporally extended goals*, i.e., goals formalized in terms of time, e.g., the exact order that a set of facts of a goal must be achieved in a plan. Recently, [1] propose a general formulation of a temporal inference problem in deterministic planning settings. However, most of these approaches also assume that the observed actions’ outcomes are deterministic and do not deal with unpredictable, possibly adversarial, environmental conditions.

Research on planning for *temporally extended goals* in *deterministic* and *non-deterministic* domains has increased over the years, starting with the pioneering work on planning for temporally extended goals [5] and on planning via model checking [12]. This continued with the work on integrating ltl goals into planning tools [27, 28], and, most recently, [7], introducing a novel optimal encoding of Pure-Past Linear Temporal Logic goals into for *Classical Planning*. Other existing works relate *program synthesis* [33] with planning in non-deterministic domains for temporal specifications, recently focusing on the *finite trace* variants of ltl [2, 9, 10, 14–16].

In this paper, we introduce the task of goal recognition in *discrete domains* that are *fully observable*, and the outcomes of actions and observations are *non-deterministic*, possibly adversarial, i.e., *Fully Observable Non-Deterministic* (fond), allowing the formalization of *temporally extended goals* using two types of temporal logic on finite traces: *Linear-time Temporal Logic* (ltl$_f$) and *Pure-Past Linear-time Temporal Logic* (ppltl) [17].

The main contribution of this paper is three-fold. First, based on the definition of *Plan Recognition as Planning* introduced in [34], we formalize *the problem of recognizing temporally extended goals* (expressed in ltl$_f$ or ppltl) in fond planning domains, handling both stochastic (i.e., strong-cyclic plans) and adversarial (i.e., strong plans) environments [2]. Second, we extend the probabilistic framework for goal recognition proposed in [35], and develop a novel *probabilistic approach* that reasons over executions of policies and returns a posterior probability distribution for the goal hypotheses. Third, we develop a *compilation approach* that generates an augmented fond planning problem by compiling temporally extended goals together with the original planning problem. This compilation allows us to use any off-the-shelf fond planner to perform the recognition task in fond planning models with temporally extended goals.

This work focuses on fond domains with stochastic non-determinism, and conduct an extensive set of experiments with different complex problems. We empirically evaluate our approach using different ltl$_f$ and ppltl goals over six fond planning domain models, including a real-world non-deterministic domain model [26], and our experiments show that our approach is accurate to recognize temporally extended goals in different two recognition settings: *offline recognition*, in which the recognition task is performed in “one-shot”, and the observations are given at once and may contain missing information; and *online recognition*, in which the observations are received incrementally, and the recognition task is performed gradually.

## Preliminaries

This section briefly recalls the syntax and semantics of *Linear-time Temporal Logics* on finite traces (ltl$_f$ and ppltl) and revises the concept and terminology of fond planning.

### ltl$_f$ and PPLTL

*Linear Temporal Logic on finite traces* (ltl$_f$) is a variant of ltl introduced in [32] interpreted over *finite traces*. Given a set of atomic propositions *AP*, the syntax of ltl$_f$ formulas $\varphi$ is defined as follows:

$$\begin{aligned} \varphi {:}{:}= a \mid \lnot \varphi \mid \varphi \wedge \varphi \mid {\circ } \varphi \mid \varphi \mathcal {U} \varphi \end{aligned}$$

where *a* denotes an atomic proposition in *AP*, $${\circ }$$ is the *next* operator, and $$\mathcal {U}$$ is the *until* operator. Apart from the Boolean connectives, we use the following abbreviations: *eventually* as $$\Diamond \varphi \doteq \textit{true} \mathcal {U}\varphi $$; *always* as $$\Box \varphi \doteq \lnot \Diamond \lnot \varphi $$; *weak next* $${{\bullet }{} }$$ $$\varphi \doteq \lnot $$ $${\circ }$$$$\lnot \varphi $$. A trace $${\tau } = {\tau }_0 {\tau }_1 \cdots $$ is a sequence of propositional interpretations, where $${\tau }_m \in 2^{AP} (m \ge 0)$$ is the *m*-th interpretation of $${\tau }$$, and $$|{\tau }|$$ is the length of $${\tau }$$. We denote a finite trace formally as $${\tau } \in (2^{AP})^*$$. Given a finite trace $${\tau }$$ and an ltl$$_f$$ formula $$\varphi $$, we inductively define when $$\varphi $$ *holds* in $${\tau }$$ at position *i* $$(0 \le i < |{\tau }|)$$, written $${\tau }, i \models \varphi $$ as follows:

- $${\tau }, i \models a \;\text {iff}\; a \in {\tau }_i$$;
- $${\tau }, i \models \lnot \varphi \;\text {iff}\; {\tau }, i \nvDash \varphi $$;
- $${\tau }, i \models \varphi _1 \wedge \varphi _2 \;\text {iff}\; {\tau }, i \models \varphi _1 \;\text {and}\; {\tau }, i \models \varphi _2$$;
- $${\tau }, i \models $$ $${\circ }$$ $$\varphi \;\text {iff}\; i+1 < |{\tau }| \text {and} {\tau },i+1 \models \varphi $$;
- ${\tau }, i \models \varphi _1 \mathcal {U} \varphi _2$ iff there exists *j* such that $i\le j < |{\tau }|$ and ${\tau },j \models \varphi _2$, and for all $k, ~i\le k < j$, we have ${\tau }, k \models \varphi _1$.

An ltl$_f$ formula $\varphi$ is *true* in ${\tau }$, denoted by ${\tau } \models \varphi$, iff ${\tau },0 \models \varphi$. As advocated by [17], this paper also uses the *pure-past* version of ltl$_f$, here denoted as ppltl, due to its compelling computational advantage compared to ltl$_f$ when goal specifications are *naturally* expressed in a past fashion. ppltl refers *only* to the past and has a natural interpretation on finite traces: formulas are satisfied if they hold in the current (i.e., last) position of the trace.

Given a set *AP* of propositional symbols, ppltl formulas are defined by:

$$\begin{aligned} \varphi {:}{:}= a \mid \lnot \varphi \mid \varphi \wedge \varphi \mid {\circleddash } \varphi \mid \varphi \mathcal {S} \varphi \end{aligned}$$

where $a\in AP$, ${\circleddash }$ is the *before* operator, and $\mathcal {S}$ is the *since* operator. Similarly to ltl$_f$, common abbreviations are the *once* operator  and the *historically* operator  . Given a finite trace ${\tau }$ and a ppltl formula $\varphi$, we inductively define when $\varphi$ *holds* in ${\tau }$ at position *i* $(0 \le i < |{\tau }|)$, written ${\tau }, i \models \varphi$ as follows. For atomic propositions and Boolean operators it is as for ltl$_f$. For past operators:

- $${\tau },i \models {\circleddash } \varphi $$ iff $$i-1 \ge 0$$ and $${\tau },i-1 \models \varphi $$;
- ${\tau },i \models \varphi _1 \mathcal {S} \varphi _2$ iff there exists *k* such that $0 \le k \le i$ and ${\tau },k \models \varphi _2$, and for all *j*, $k<j\le i$, we have ${\tau },j \models \varphi _1$.

A ppltl formula $\varphi$ is *true* in ${\tau }$, denoted by ${\tau } \models \varphi$, if and only if ${\tau }, |{\tau }|-1 \models \varphi$. A key property of temporal logics exploited in this work is that, for every ltl$_f$/ppltl formula $\varphi$, there exists a *Deterministic Finite-state Automaton* (DFA) $\mathcal {A}_{\varphi }$ accepting the traces ${\tau }$ satisfying $\varphi$  [15, 17].

**Fig. 1:**
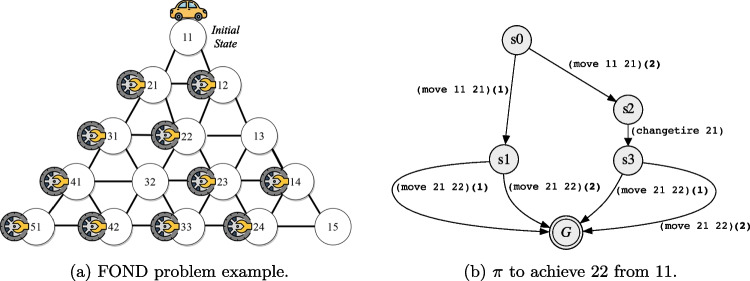
Triangle-Tireworld domain and policy

### fond planning

A *Fully Observable Non-deterministic Domain* planning model (fond) is a tuple $\mathcal {D} = {\langle {2^{\mathcal {F}}, A, \alpha , tr}\rangle }$ [18], where $2^{\mathcal {F}}$ is the set of possible states and $\mathcal {F}$ is a set of fluents (atomic propositions); *A* is the set of actions; $\alpha (s) \subseteq A$ is the set of applicable actions in a state *s*; and *tr*(*s*, *a*) is the non-empty set of successor states that follow action *a* in state *s*. A domain $\mathcal {D}$ is assumed to be compactly represented (e.g., in PDDL [24]), hence its size is $|\mathcal {F}|$. Given the set of *literals* of $\mathcal {F}$ as $\textit{Literals}(\mathcal {F}) = \mathcal {F} \cup \{ \lnot f \mid f \in \mathcal {F} \}$, every action $a \in A$ is usually characterized by ${\langle {{ Pre }_a, {\textit{Eff}}_a}\rangle }$, where ${ Pre }_a \subseteq \textit{Literals}(\mathcal {F})$ is the action preconditions, and ${\textit{Eff}}_a$ is the action effects. An action *a* can be applied in a state *s* if the set of fluents in ${ Pre }_a$ holds true in *s*. The result of applying *a* in *s* is a successor state $s'$ non-deterministically drawn from one of the ${\textit{Eff}}^{i}_{a}$ in ${\textit{Eff}}_a = \{ {\textit{Eff}}^{1}_{a}, ..., {\textit{Eff}}^{n}_{a} \}$. In fond planning, some actions have *uncertain outcomes*, such that they have *non-deterministic* effects (i.e., $|tr(s, a)| \ge 1$ in all states *s* in which *a* is applicable), and effects cannot be predicted in advance. PDDL expresses uncertain outcomes using the oneof [8] keyword, as widely used by several fond planners [23, 25]. A fond planning problem is formally defined as follows.

#### Definition 1

A fond **planning problem** is a tuple $\mathcal {P} = {\langle {\mathcal {D}, s_{0}, G}\rangle }$, where $\mathcal {D}$ is a fond domain model, $s_{0}$ is an initial assignment to fluents in $\mathcal {F}$ (i.e., initial state), and $G \subseteq \mathcal {F}$ is the goal.

Solutions to a fond planning problem $\mathcal {P}$ are *policies*. A policy is usually denoted as $\pi$, and formally defined as a partial function $\pi : 2^{\mathcal {F}} \rightarrow A$ mapping *non-goal* states into applicable actions that eventually reach a goal state complying with *G* from the initial state $s_{0}$. We say a state *s* complies with *G* if $G \subseteq s$. A *policy* $\pi$ for $\mathcal {P}$ induces a set of possible *executions* $\vec {E} = \{ \vec {e}_1, \vec {e}_2, \dots \}$, that are state trajectories, possibly finite (i.e., histories) $(s_{0},\dots , s_{n})$, where $s_{i+1} \in tr(s_i, a_i)$ and $a_i \in \alpha (s_i)$ for $i = 0,\dots , n-1$, or possibly infinite $s_{0},s_{1},\dots$, obtained by choosing some possible outcome of actions instructed by the policy. A policy $\pi$ is a solution to $\mathcal {P}$ if every execution is finite and satisfies the goal *G* in its last state, i.e., $s_{n} \models G$. In this case, $\pi$ is *winning*. [13] define three solutions to fond planning problems: *weak, strong* and *strong-cyclic* solutions, formally defined in Definitions 2, 4, and 3.

#### Definition 2

A **weak solution** is a policy that achieves a goal state complying with *G* from the initial state $s_{0}$ under at least one selection of action outcomes; namely, such solution will have some chance of achieving a goal state complying with *G*.

#### Definition 3

A **strong-cyclic solution** is a policy that guarantees to achieve a goal state complying with *G* from the initial state $s_{0}$ only under the assumption of fairness^1^. However, this type of solution may revisit states, so the solution cannot guarantee to achieve *G* in a fixed number of steps.

#### Definition 4

A **strong solution** is a policy that is guaranteed to achieve a goal state complying with *G* from the initial state $s_{0}$ regardless of the environment’s non-determinism. This type of solution guarantees the achievement of *G* in a finite number of steps while never visiting the same state twice.

This work focuses on *strong-cyclic solutions*, where the environment acts in an unknown but stochastic way. Nevertheless, our recognition approach applies to strong solutions as well, where the environment is purely adversarial (i.e., the environment may always choose effects against the agent).

Our running example comes from the well-known Triangle-Tireworld fond domain, where roads connect locations, and the agent can drive through them. The objective is to drive from one location to another. However, while driving between locations, a tire may go flat, and if there is a spare tire in the car’s location, then the car can use it to fix the flat tire. Figure 1a illustrates a fond planning problem for the Triangle-Tireworld domain, where circles are locations, arrows represent roads, spare tires are depicted as tires, and the agent is depicted as a car. Figure 1b shows a policy $\pi$ to achieve location 22. Note that, to move from location 11 to location 21, there are two arrows labeled with the action (move 11 21): (1) when moving does not cause the tire to go flat; (2) when moving causes the tire to go flat. The policy depicted in Fig. 1b guarantees the success of achieving location 22 despite the environment’s non-determinism.

From *Classical Planning*, the cost for all *non-deterministic* instantiated actions $a \in A$ is 1. In this example, policy $\pi$, depicted in Fig. 1b, has two possible finite executions in the set of executions $\vec {E}$, namely $\vec {E} = \{ \vec {e}_{0}, \vec {e}_{1} \}$, such as:

- $\vec {e}_{0}$: [(move 11 21), (move 21 22)]; and
- $\vec {e}_{1}$: [(move 11 21), (changetire 21), (move 21 22)].

## FOND planning for ltl$_f$ and PPLTL goals

We base our approach to goal recognition in fond domains for *temporally extended goals* on fond planning with ltl$_f$ and ppltl goals [9, 10, 14]. Definition 5 formalizes a fond planning problem with ltl$_f$/ppltl goals as follows.

### Definition 5

A fond **planning problem with** ltl$_f$/ppltl **goals** is a tuple $\Gamma = {\langle {\mathcal {D}, s_0, \varphi }\rangle }$, where $\mathcal {D}$ is a standard fond domain model, $s_0$ is the initial state, and $\varphi$ is a goal formula, formally represented either as an ltl$_f$ or a ppltl formula.

In fond planning with temporally extended goals, a policy $\pi$ is a partial function $\pi : (2^\mathcal {F})^{+} \rightarrow A$ mapping *histories*, i.e., *states* into applicable actions. A policy $\pi$ for $\Gamma$ achieves a temporal formula $\varphi$ if and only if the sequence of states generated by $\pi$, despite the non-determinism of the environment, is accepted by $\mathcal {A}_\varphi$.

Key to our recognition approach is encoding the temporal goal formula into an extended planning domain, expressed in PDDL, which can be later consumed by off-the-shelf fond planners. Compiling planning for temporally extended goals into planning for standard *reachability* goals (i.e., final-state goals) has a long history in the AI *Planning* literature. In particular, [6] develops *deterministic* planning with special first-order quantified ltl goals on finite-state sequences. Their technique encodes a *Non-Deterministic Finite-state Automaton* (NFA), resulting from ltl formulas, into deterministic planning domains for which *Classical Planning* technology can be leveraged. Our parameterization of objects of interest is somehow similar to their approach. Starting from [6], always in the context of deterministic planning, [38] proposed a polynomial-time compilation of ltl goals on finite-state sequences into alternating automata, leaving non-deterministic choices to be decided at planning time. Finally, [9, 10] built upon [6] and [38], proposing a compilation in the context of fond domain models that explicitly computes the automaton representing the ltl$_f$ temporal goal and encodes it into PDDL. However, this encoding introduces a lot of bookkeeping machinery due to the removal of any form of angelic non-determinism mismatching with the devilish non-determinism of PDDL for fond.

Although inspired by such work, our approach differs in several technical details. We encode the DFA directly into a non-deterministic PDDL planning domain by taking advantage of the *parametric* nature of PDDL domains that are then instantiated into propositional problems when solving a specific task. Given a fond planning problem $\Gamma$ represented in PDDL, the transformation $\Gamma$ works as follows. First, the highly-optimized MONA tool [20] transforms the temporally extended goal formula $\varphi$ (formalized either in ltl$_f$ or ppltl) into its corresponding DFA $\mathcal {A}_\varphi$. Second, from $\mathcal {A}_\varphi$, we build a *parametric* DFA (PDFA), representing the lifted version of the DFA. Finally, the encoding of such a PDFA into PDDL yields an augmented fond domain model $\Gamma '$. Thus, this process reduces fond planning for ltl$_f$/ppltl to a standard fond planning problem solvable by any off-the-shelf fond planner.

### Translation to parametric DFA

The use of *parametric* DFAs is based on the following observations. In temporal logic formulas and, hence, in the corresponding DFAs, propositions are represented by domain fluents grounded on specific objects of interest. We can replace these propositions with predicates using object variables and then have a mapping function $m^{obj}$ that maps such variables into the problem instance objects. This yields a lifted and *parametric* representation of the DFA, i.e., PDFA, which is merged with the domain. Here, the objective is to capture the entire dynamics of the DFA within the planning domain model itself. To do so, starting from the DFA we build a PDFA whose states and symbols are the lifted versions of the ones in the DFA. Formally, to construct a PDFA we use a mapping function $m^{obj}$, which maps the set of objects of interest present in the DFA to a set of *free* variables. Given the mapping function $m^{obj}$, Definition 6 formalizes a PDFA as follows.

#### Definition 6

Given a set of object symbols $\mathcal {O}$, and a set of free variables $\mathcal {V}$, we define a **mapping function** *m* that maps each object in $\mathcal {O}$ with a free variable in $\mathcal {V}$.

Given a DFA and the objects of interest for $\Gamma$, we can construct a PDFA as follows:

#### Definition 7

A PDFA is a tuple $\mathcal {A}^{p}_\varphi = {\langle {\Sigma ^{p}, Q^{p}, q^{p}_0, \delta ^{p}, F^{p}}\rangle }$, where: $\Sigma ^{p} = \{ \sigma ^p_0, ..., \sigma ^p_n \} = 2^{\mathcal {F}}$ is the alphabet of fluents; $Q^{p}$ is a nonempty set of parametric states; $q^{p}_0$ is the parametric initial state; $\delta ^{p}: Q^{p} \times \Sigma ^{p} \rightarrow Q^{p}$ is the parametric transition function; $F^{p} \subseteq Q^{p}$ is the set of parametric final states. $\Sigma ^{p}, Q^{p}, q^{p}_0, \delta ^{p}$ and $F^{p}$ can be obtained by applying $m^{obj}$ to all the components of the corresponding DFA.

#### Example 1

**Fig. 2:**
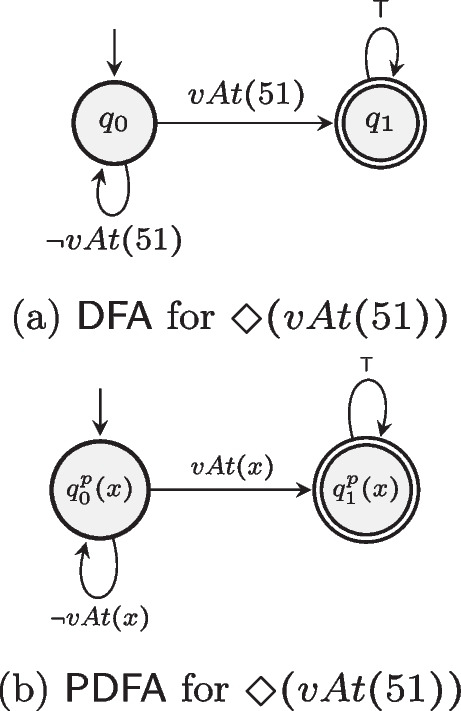
DFA and PDFA for $\Diamond (vAt(51))$

Given the ltl$_f$ formula “$\Diamond (vAt ~51)$”, the object of interest “51” is replaced by the object variable *x* (i.e., $m^{obj}(51) = x$), and the corresponding DFA and PDFA for this ltl$_f$ formula are depicted in Fig. 2a and b.

When the resulting new domain is instantiated, we implicitly get back the original DFA in the Cartesian product with the original instantiated domain. Note that this way of proceeding is similar to what is done in [6], where they handle ltl$_f$ goals expressed in a special fol syntax, with the resulting automata (non-deterministic Büchi automata) parameterized by the variables in the ltl$_f$ formulas.

### PDFA encoding in PDDL

Once the PDFA has been computed, we encode its components within the planning problem $\Gamma$, specified in PDDL, thus, producing an augmented fond planning problem $\Gamma ' = {\langle {\mathcal {D}', s'_0, G'}\rangle }$, where $\mathcal {D}' = {\langle {2^{\mathcal {F}'}, A', \alpha ', tr'}\rangle }$ and $G'$ is a propositional goal as in *Classical Planning*. Intuitively, additional parts of $\Gamma '$ are used to synchronize the dynamics between the domain and the automaton sequentially. Specifically, $\Gamma '$ is composed of the following components.

#### Fluents

$\mathcal {F}'$ has the same fluents in $\mathcal {F}$ plus fluents representing each state of the PDFA, and a fluent called turnDomain, which controls the alternation between domain’s actions and the PDFA’s synchronization action. Formally, $\mathcal {F}' = \mathcal {F} \cup \{ q \mid q \in Q^p \} \cup \{\texttt {turnDomain} \}$.

#### Domain actions

Actions in *A* are modified by adding turnDomain in preconditions and the negated turnDomain in effects: ${ Pre }^\prime _a = { Pre }_a \cup \{ \texttt {turnDomain} \}$ and ${\textit{Eff}}^\prime _a = {\textit{Eff}}_a \cup$ $\{ \lnot \texttt {turnDomain} \}$ for all $a \in A$.

#### Transition operator

The *transition* function $\delta ^{p}$ of a PDFA is encoded as a new domain operator with conditional effects, called trans. Namely, ${ Pre }_{\texttt {trans}} = \{\lnot \texttt {turnDomain} \}$ and ${\textit{Eff}}_{\texttt {trans}} = \{ \texttt {turnDomain} \} \cup \{\texttt {{\textbf {when}}}~ (q^p, \sigma ^p), \texttt {{\textbf {then}}}~ \delta ^p(q^p, \sigma ^p) \cup \{ \lnot q \mid q \ne q^p, q \in Q^p \} \}$, for all $(q^p, \sigma ^p) \in \delta ^p$. To exemplify how the transition PDDL operator is obtained, Listing 1 reports the transition operator for the PDFA in Fig. 2.
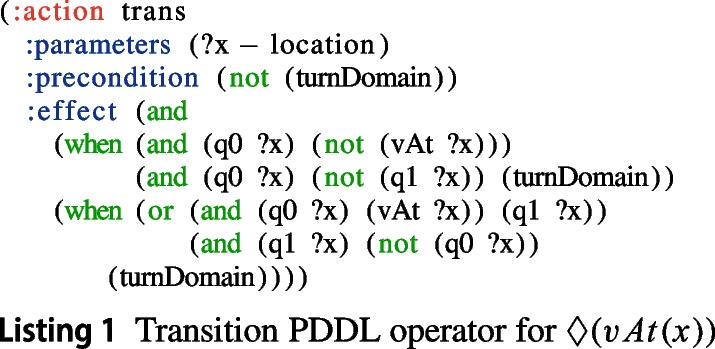


#### Initial and goal states

The new initial condition is specified as $s'_0 = s_0 \cup \{ q^{p}_0 \} \cup \{\texttt {turnDomain} \}$. This comprises the initial condition of the previous domain *D* ($s_0$) plus the initial state of the PDFA and the predicate turnDomain. Considering the example in Fig. 1a and the PDFA in Fig. 2b, the new initial condition is as follows in PDDL:
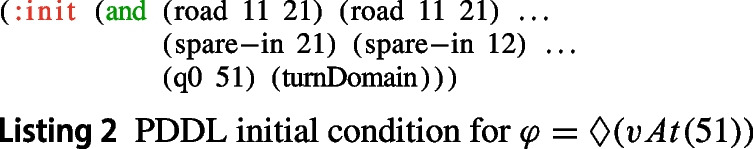


The new goal condition is specified as $G' = \{ \bigvee q_i \mid q_i \in F^{p} \} \cup \{\texttt {turnDomain} \}$, i.e., we want the PDFA to be in one of its accepting states and turnDomain, as follows:
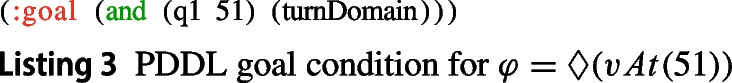


Note that, both in the initial and goal conditions of the new planning problem, PDFA states are grounded back on the objects of interest thanks to the inverse of the mapping $m^{obj}$.

**Fig. 3:**
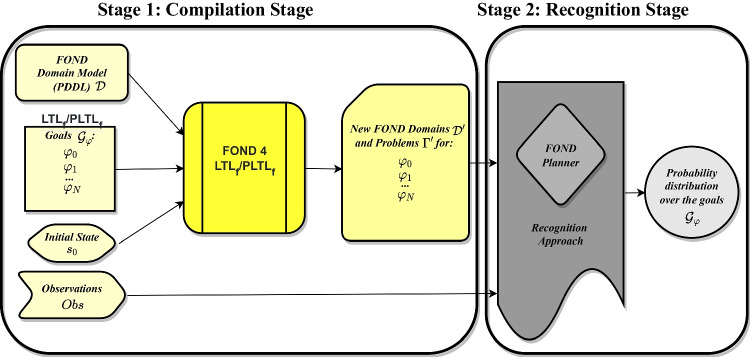
Overview of our solution approach

Executions of a policy for our new fond planning problem $\Gamma '$ are $\vec {e}': [a'_1, t_1, a'_2, t_2, \dots , a'_n,t_n]$, where $a'_i \in A'$ are the real domain actions, and $t_1, \dots , t_n$ are sequences of synchronization trans actions, which, at the end, can be easily removed to extract the desired execution $\vec {e} :[a'_1, a'_2, \dots , a'_n]$. In the remainder of the paper, we refer to the compilation just exposed as fondforLTLPLTL.

#### Theoretical property of the PDDL encoding

We now study the theoretical properties of the encoding presented in this section. Theorem 1 states that solving fond planning for ltl$_f$/ppltl goals amounts to solving standard fond planning problems for reachability goals. A policy for the former can be easily derived from a policy for the latter.

##### Theorem 1

Let $\Gamma$ be a fond planning problem with an ltl$_f$/ppltl goal $\varphi$, and $\Gamma ^\prime$ be the compiled fond planning problem with a reachability goal. Then, $\Gamma$ has a policy $\pi : (2^\mathcal {F})^+ \rightarrow A$ iff $\Gamma ^\prime$ has a policy $\pi ^\prime : (2{^\mathcal {F}}^\prime )^+ \rightarrow A^\prime$.

##### Proof

($\longrightarrow$). We start with a policy $\pi$ of the original problem that is winning by assumption. Given $\pi$, we can always build a new policy, which we call $\pi ^\prime$, following the encoding presented in Section 3 of the paper. The newly constructed policy will modify histories of $\pi$ by adding fluents and an auxiliary deterministic action $\texttt {trans}$, both related to the DFA associated with the ltl$_f$/ppltl formula $\varphi$. Now, we show that $\pi ^\prime$ is an executable policy and that is winning for $\Gamma ^\prime$. To see the executability, observe that, by construction of the new planning problem $\Gamma ^\prime$, all action effects ${\textit{Eff}}_{a^\prime }$ of the original problem $\Gamma$ are modified in a way that all action effects of the original problem $\Gamma$ are not modified and that the auxiliary action $\texttt {trans}$ only changes the truth value of additional fluents given by the DFA $\mathcal {A}^p_\varphi$ (i.e., automaton states). Therefore, the newly constructed policy $\pi ^\prime$ can be executed. To see that $\pi ^\prime$ is winning and satisfies the ltl$_f$/ppltl goal formula $\varphi$, we reason about all possible executions. For all executions, every time the policy $\pi ^\prime$ stops we can always extract an induced state trajectory of length *n* such that its last state $s^\prime _n$ will contain one of the final states $F^p$ of the automaton $\mathcal {A}^p_\varphi$. This means that the induced state trajectory is accepted by the automaton $\mathcal {A}^p_\varphi$. Then, by Theorem [15, 17] ${\tau }\models \varphi$.

($\longleftarrow$). From a winning policy $\pi ^\prime$ for the compiled problem, we can always project out all automata auxiliary $\texttt {trans}$ actions obtaining a corresponding policy $\pi$. We need to show that the resulting policy $\pi$ is winning, namely, it can be successfully executed on the original problem $\Gamma$ and satisfies the ltl$_f$/ppltl goal formula $\varphi$. The executability follows from the fact that the deletion of $\texttt {trans}$ actions and related auxiliary fluents from state trajectories induced by $\pi$ does not modify any precondition/effect of original domain actions (i.e., $a\in \mathcal {A}$). Hence, under the right preconditions, any domain action can be executed. Finally, the satisfaction of the ltl$_f$/ppltl formula $\varphi$ follows directly from Theorem [15, 17]. Indeed, every execution of the winning policy $\pi ^\prime$ stops when reaching one of the final states $F^p$ of the automaton $\mathcal {A}^p_\varphi$ in the last state $s_n$, thus every execution of $\pi$ would satisfy $\varphi$. Thus, the thesis holds. $\square$

## Goal recognition in fond planning domains with ltl$_f$ and PPLTL goals

This section introduces the recognition approach that is able to recognizing temporally extended (ltl$_f$ and ppltl) goals in fond planning domains. Our approach extends the probabilistic framework of [35] to compute posterior probabilities over temporally extended goal hypotheses, by reasoning over the set of possible executions of policies $\pi$ and the observations. This works in two stages: the *compilation stage* and the *recognition stage*. The following sections describe in detail how these two stages work. Figure 3 illustrates how our approach works.

### Goal recognition problem

We define the task of goal recognition in fond planning domains with ltl$_f$ and ppltl goals by extending the standard definition of *Plan Recognition as Planning* [34], as follows.

#### Definition 8

A **temporally extended goal recognition problem** in a fond planning setting with temporally extended goals (ltl$_f$ and/or ppltl) is a tuple $\mathcal {T}_{\varphi } = \langle \mathcal {D}, s_0, \mathcal {G}_{\varphi }, Obs\rangle$, where: $\mathcal {D} = {\langle {2^{\mathcal {F}}, A, \alpha , tr}\rangle }$ is a fond planning domain; $s_0$ is the initial state; $\mathcal {G}_{\varphi } = \lbrace \varphi _0, \varphi _1, ..., \varphi _n \rbrace$ is the set of goal hypotheses formalized in ltl$_f$ or ppltl, including the intended goal $\varphi ^{*} \in \mathcal {G}_{\varphi }$; $Obs = {\langle {o_0, o_1, ..., o_n}\rangle }$ is a sequence of successfully executed (non-deterministic) actions of a policy $\pi _{\varphi ^{*}}$ that achieves the intended goal $\varphi ^{*}$, s.t. $o_i \in A$.

Since we deal with non-deterministic domain models, an observation sequence *Obs* corresponds to a successful execution $\vec {e}$ in the set of all possible executions $\vec {E}$ of a *strong-cyclic policy* $\pi$ that achieves the actual intended hidden goal $\varphi ^{*}$. In this work, we assume two recognition settings: *Offline Keyhole Recognition*, and *Online Recognition*. In *Offline Keyhole Recognition* the observed agent is completely unaware of the recognition process [3], the observation sequence *Obs* is given at once, and it can be either *full* or *partial*—in a *full observation sequence*, the recognizer has access to all actions of an agent’s plan, whereas, in a *partial observation sequence*, only a sub-sequence thereof. By contrast, in *Online Recognition* [39], the observed agent is also unaware of the recognition process, but the observation sequence is revealed incrementally instead of being given in advance and at once, as in *Offline Recognition*, thus making the recognition process an already much harder task.

An “ideal” solution for a goal recognition problem comprises a selection of the goal hypotheses containing only the single actual intended hidden goal $\varphi ^{*} \in \mathcal {G}$ that the observation sequence *Obs* of a plan execution achieves [34, 35]. Fundamentally, there is no exact solution for a goal recognition problem, but it is possible to produce a probability distribution over the goal hypotheses and the observations, so that the goals that “best” explain the observation sequence are the most probable ones. A solution to a goal recognition problem in fond planning with temporally extended goals is defined in Definition 9.

#### Definition 9

Solving a goal recognition problem $\mathcal {T}_{\varphi }$ requires selecting a temporally extended goal hypothesis $\hat{\varphi } \in \mathcal {G}_{\varphi }$ such that $\hat{\varphi } = \varphi ^{*}$, and it represents how well $\hat{\varphi }$ predicts or explains what observation sequence *Obs* aims to achieve.

Existing recognition approaches often return either a probability distribution over the set of goals [35, 37], or scores associated with each possible goal hypothesis [31]. Our framework returns a probability distribution $\mathbb {P}$ over the set of temporally extended goals $\mathcal {G}_{\varphi }$ that “best” explains the observations sequence *Obs*.

### Probabilistic goal recognition

The probabilistic framework for *Plan Recognition as Planning* of [35] sets the probability distribution for every goal *G* in the set of goal hypotheses $\mathcal {G}$, and the observation sequence *Obs* to be a Bayesian posterior conditional probability, as follows:

1 $$\begin{aligned} \mathbb {P}(G \mid Obs) = \eta * \mathbb {P}(Obs \mid G) * \mathbb {P}(G) \end{aligned}$$

where $\mathbb {P}(G)$ is the *a priori* probability assigned to goal *G*, $\eta$ is a normalization factor inversely proportional to the probability of *Obs*, and $\mathbb {P}(Obs \mid G)$ is

2 $$\begin{aligned} \mathbb {P}(Obs \mid G) = \sum _{\pi } \mathbb {P}(Obs \mid \pi ) * \mathbb {P}(\pi \mid G) \end{aligned}$$

$\mathbb {P}(Obs \mid \pi )$ is the probability of obtaining *Obs* by executing a policy $\pi$ and $\mathbb {P}(\pi \mid G)$ is the probability of an agent pursuing *G* to select $\pi$. What follows extends the probabilistic framework above to recognize temporally extended goals in fond planning domain models.

### Compilation stage

We perform a *compilation stage* that allows us to use any off-the-shelf fond planner to extract policies for temporally extended goals. To this end, we compile and generate new fond planning domain models $\Gamma '$ for the set of possible temporally extended goals $\mathcal {G}_{\varphi }$ using the compilation approach described in Section 3. Specifically, for every goal $\varphi \in \mathcal {G}_{\varphi }$, our compilation takes as input a fond planning problem $\Gamma$, where $\Gamma$ contains the fond planning domain $\mathcal {D}$ along with an initial state $s_0$ and a temporally extended goal $\varphi$. Finally, as a result, we obtain a new fond planning problem $\Gamma '$ associated with the new domain $\mathcal {D}'$. Note that such a new fond planning domain $\Gamma '$ encodes new predicates and transitions that allow us to plan for temporally extended goals by using off-the-shelf fond planners.

#### Corollary 1

Let $\mathcal {T}_\varphi$ be a goal recognition problem over a set of ltl$_f$/ppltl goals $\mathcal {G}_\varphi$ and let $\mathcal {T^\prime }$ be the compiled goal recognition problem over a set of propositional goals $\mathcal {G}$. Then, if $\mathcal {T^\prime }$ has a set of winning policies that solve the set of propositional goals in $\mathcal {G}$, then $\mathcal {T}_\varphi$ has a set of winning policies that solve its ltl$_f$/ppltl goals.

#### Proof

It follows from Theorem 1 that a bijective mapping exists between policies of fond planning for ltl$_f$/ppltl goals and policies of standard fond planning. Therefore, the thesis holds. $\square$

### Recognition stage

The stage that performs the goal recognition task comprises extracting policies for every goal $\varphi \in \mathcal {G}_{\varphi }$. From such policies along with observations *Obs*, we compute posterior probabilities for the goals $\mathcal {G}_{\varphi }$ by matching the observations with all possible executions in the set of executions $\vec {E}$ of the policies. To ensure compatibility with the policies, the recognizer assumes knowledge of the preference relation over actions for the observed agent when unrolling the policy during search.

#### Computing policies and the set of executions $\vec {E}$ for $\mathcal {G}_{\varphi }$

The recognizer extracts policies for every goal $\varphi \in \mathcal {G}_{\varphi }$ using the new fond planning domain models $\Gamma '$, and for each of these policies, it enumerates the set of possible executions $\vec {E}$. The aim of enumerating the possible executions $\vec {E}$ for a policy $\pi$ is to attempt to infer what execution $\vec {e} \in \vec {E}$ the observed agent is performing in the environment. Environmental non-determinism prevents the recognizer from determining the specific execution $\vec {e}$ the observed agent goes through to achieve its goals. The recognizer considers possible executions that are all paths to the goal with no repeated states. The fact that the probability of entering loops multiple times is low partially justifies this assumption, and relaxing it is an important research direction for future work.

After enumerating the set of possible executions $\vec {E}$ for a policy $\pi$, we compute the average distance of all actions in the set of executions $\vec {E}$ to a goal $\varphi$ from initial state $s_{0}$. Note that strong-cyclic solutions may have infinite possible executions. However, here we consider executions that do not enter loops, and for those entering possible loops, we consider only the ones entering loops *at most* once. Indeed, the occurrence of possibly repeated actions does not affect the computation of the average distance. In other words, if the observed agent executes the same action repeatedly often, it does not change its distance to the goal. The average distance aims to estimate “how far” every observation $o \in Obs$ is to goal $\varphi$. This average distance is computed because some executions $\vec {e} \in \vec {E}$ may share the same action in execution sequences but at different time steps. We refer to this average distance as $\textbf{d}$. For example, consider the policy $\pi$ depicted in Fig. 1b. This policy $\pi$ has two possible executions for achieving a goal from the initial state, and these two executions share some actions, such as (move 11 21). In particular, this action appears twice in Fig. 1b due to its uncertain outcome. Therefore, this action has two different distances (if we count the number of remaining actions towards a goal) to the goal: $distance = 1$, if the outcome of this action generates the state $s_2$; and $distance = 2$, if the outcome of this action generates the state $s_3$. Hence, since this policy $\pi$ has two possible executions, and the sum of the distances is 3, the average distance for this action to a goal is $\textbf{d} = 1.5$. The average distances for the other actions in this policy are: $\textbf{d} = 1$ for (changetire 21), because it appears only in one execution; and $\textbf{d} = 0$ for (move 21 22), because the execution of this action achieves a goal.

We use $\textbf{d}$ to compute an *estimated score* that expresses “how far” every observed action in the observation sequence *Obs* is to a temporally extended goal $\varphi$ in comparison to the other goals in the set of goal hypotheses $\mathcal {G}_{\varphi }$. This means that the goal(s) with the lowest score(s) along the execution of the observed actions $o \in Obs$ is (are) the one(s) that, most likely, the observation sequence *Obs* aims to achieve. Note that, the average distance $\textbf{d}$ for those observations $o \in Obs$ that are not in the set of executions $\vec {E}$ of a policy $\pi$, is set to a large constant number, i.e., to $\textbf{d} = e^{5}$. As part of the computation of this *estimated score*, we compute a *penalty value* that directly affects the *estimated score*. This *penalty value* represents a penalization that aims to increase the *estimated score* for those goals in which each pair of subsequent observations $\langle o_{i-1}, o_{i} \rangle$ in *Obs* does not have any relation of order in the set of executions $\vec {E}$ of these goals. We use the Euler constant *e* to compute this *penalty value*, formally defined as $e^{\textbf{p}(o_{i-1}, o_{i})}$, in which $\mathcal {R}(\vec {e})$ is the set of order relation of an execution $\vec {e}$, where

3 $$\begin{aligned} \textbf{p}(o_{i-1}, o_{i}) = {\left\{ \begin{array}{ll} 1, &{} \text {if } \lbrace \forall \vec {e} \in E | \langle o_{i-1} \prec o_{i} \rangle \notin \mathcal {R}(\vec {e})\rbrace \\ 0, &{} \text {otherwise} \end{array}\right. } \end{aligned}$$

Equation (4) formally defines the computation of the *estimated score* for every goal $\varphi \in \mathcal {G}_{\varphi }$ given a pair of subsequent observations $\langle o_{i-1}, o_{i} \rangle$, and the set of goal hypotheses $\mathcal {G}_{\varphi }$.

4 $$\begin{aligned} {\frac{{e^{\textbf{p}(o_{i-1}, o_{i})}}{*}\textbf{d}(o_{i}, \varphi )}{\sum _{\varphi ' \in \mathcal {G}_{\varphi }} \textbf{d}(o_{i}, \varphi ')}} \end{aligned}$$

##### Example 2

**Fig. 4:**
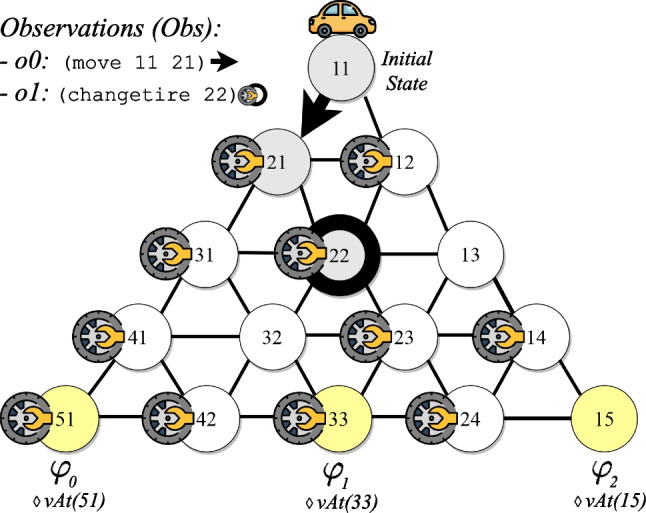
Recognition problem example

To exemplify the computation of the *estimated score* for every goal $\varphi \in \mathcal {G}_{\varphi }$, consider the recognition problem in Fig. 4: $s_0$ is *vAt*(11); the goal hypotheses $\mathcal {G}_{\varphi }$ are expressed as ltl$_f$ goals, such that $\varphi _0 = \Diamond vAt(51), \varphi _1 = \Diamond vAt(33)$, and $\varphi _2 = \Diamond vAt(15)$; $Obs = \lbrace o_0: { \texttt {(move 11 21)}}, o_1: { \texttt {(changetire 22)}} \rbrace$. The intended goal $\varphi ^{*}$ is $\varphi _1$. Before computing the *estimated score* for the goals, we first perform the compilation process presented before. Afterward, we extract policies for every goal $\varphi \in \mathcal {G}_{\varphi }$, enumerate the possible executions $\vec {E}$ for the goals $\mathcal {G}_{\varphi }$ from the extracted policies, and then compute the average distance $\textbf{d}$ of all actions in the set of executions $\vec {E}$ for the goals $\mathcal {G}_{\varphi }$ from $s_{0}$. The number of possible executions $\vec {E}$ for the goals are: $\varphi _0: |\vec {E}| = 8, \varphi _1: |\vec {E}| = 8$, and $\varphi _2 = |\vec {E}| = 16$. The average distances $\textbf{d}$ of all actions in $\vec {E}$ for the goals are as follows:

- $\varphi _0$: (move 11 21) = 4.5, (changetire 21) = 4, (move 21 31) = 3, (changetire 31) = 2.5, (move 31 41) = 1.5, (changetire 41) = 1, (move 41 51) = 0;
- $\varphi _1$: (move 11 21) = 4.5, (changetire 21) = 4, (move 21 22) = 3, (changetire 22) = 2.5, (move 22 23) = 1.5, (changetire 23) = 1, (move 23 33): 0;
- $\varphi _2$: (move 11 21) = 6, changetire 21) = 5.5, (move 21 22) = 4.5, (changetire 22) = 4, (move 22 23) = 3, (changetire 23) = 2.5, (changetire 24) = 1, (move 23 24) = 1.5, (move 24 15) = 0.

Once having the average distances $\textbf{d}$ of the actions in $\vec {E}$ for all goals, we can then compute the *estimated score* for $\mathcal {G}_{\varphi }$ for every observation $o \in Obs$: $o_0 \texttt {(move 11 21)}: \varphi _0 = \frac{4.5}{4.5 + 6} =$ 0.43, $\varphi _1 = \frac{4.5}{4.5 + 6} =$ 0.43, $\varphi _2 = \frac{6}{4.5 + 6} =$ 0.57; and $o_1 \texttt {(changetire 22)}: \varphi _0 = \frac{e^1 * e^5}{6.5} =$ 61.87, $\varphi _1 = \frac{2.5}{e^5 + 2.5} =$ 0.016, $\varphi _2 = \frac{4}{e^5 + 4} =$ 0.026. Note that for the observation $o_1$, the average distance $\textbf{d}$ for $\varphi _0$ is $e^5 = 148.4$ because this observation is not an action for one of the executions in the set of executions for this goal (*Obs* aims to achieve the intended goal $\varphi ^{*} = \varphi _1$). Furthermore, the *penalty value* is applied to $\varphi _0$, i.e., $e^1 = 2.71$. It is possible to see that the *estimated score* of the intended goal $\varphi _1$ is always the lowest for all observations *Obs*, especially when observing the second observation $o_1$. Note that our approach correctly infers the intended goal $\varphi ^{*}$, even when observing with just few actions.

#### Computing posterior probabilities for $\mathcal {G}_{\varphi }$

To compute the posterior probabilities over the set of possible temporally extended goals $\mathcal {G}_{\varphi }$, we start by computing the *average estimated score* for every goal $\varphi \in \mathcal {G}_{\varphi }$ for every observation $o \in Obs$, and we formally define this computation as $\mathcal {E}(\varphi , Obs, \mathcal {G}_{\varphi })$, as follows:

5 $$\begin{aligned} \mathcal {E}(\varphi , Obs, \mathcal {G}_{\varphi }) = \left( \frac{\sum \limits _{i=0}^{|Obs|} \frac{{e^{\textbf{p}(o_{i-1}, o_{i})}}{*} \varvec{d}(o_{i}, \varphi )}{\sum _{\varphi ' \in \mathcal {G}_{\varphi }} \varvec{d}(o_{i}, \varphi ')}}{|Obs|} \right) \end{aligned}$$

The *average estimated score* $\mathcal {E}$ aims to estimate “how far” a goal $\varphi$ is to be achieved compared to other goals ($\mathcal {G}_{\varphi } \setminus \{\varphi \}$) *averaging* among all the observations in *Obs*. The lower the *average estimated score* $\mathcal {E}$ to a goal $\varphi$, the more likely such a goal is to be the one that the observed agent aims to achieve. Consequently, $\mathcal {E}$ has two important properties defined in (5), as follows.

##### Proposition 1

Given that the sequence of observations *Obs* corresponds to an execution $\vec {e} \in \vec {E}$ that aims to achieve the actual intended hidden goal ${\varphi }^{*} \in \mathcal {G}_{\varphi }$, the *average estimated score* outputted by $\mathcal {E}$ will tend to be the lowest for ${\varphi }^{*}$ in comparison to the scores of the other goals ($\mathcal {G}_{\varphi } \setminus \{ {\varphi }^{*} \}$), as observations increase in length.

##### Proposition 2

If we restrict the recognition setting and define that the goal hypotheses $\mathcal {G}_{\varphi }$ are not sub-goals of each other, and observe all observations in *Obs* (i.e., full observability), we will have the intended goal ${\varphi }^{*}$ with the lowest score among all goals, i.e., $\forall \varphi \in \mathcal {G}_{\varphi }$ is the case that $\mathcal {E}({\varphi }^{*}, Obs, \mathcal {G}_{\varphi }) \le \mathcal {E}(\varphi , Obs, \mathcal {G}_{\varphi })$.

After defining the computation of the *average estimated score* $\mathcal {E}$ for the goals using (5), we can define how our approach tries to maximize the probability of observing a sequence of observations *Obs* for a given goal $\varphi$, as follows:

6 $$\begin{aligned} \mathbb {P}(Obs \mid \varphi ) = [1 + \mathcal {E}(\varphi , Obs, \mathcal {G}_{\varphi })]^{-1} \end{aligned}$$

Thus, by using the *estimated score* in (6), we can infer that the goals $\varphi \in \mathcal {G}_{\varphi }$ with the lowest *estimated score* will be the most likely to be achieved according to the probability interpretation from (5). For instance, consider the goal recognition problem presented in Example 2, and the *estimated scores* we computed for the temporally extended goals $\varphi _0$, $\varphi _1$, and $\varphi _2$ based on the observation sequence *Obs*. From this, we have the following probabilities $\mathbb {P}(Obs \mid \varphi )$ for the goals:

- $$\mathbb {P}(Obs \mid \varphi _0) = [1 + (31.15)]^{-1} = 0.03$$
- $$\mathbb {P}(Obs \mid \varphi _1) = [1 + (0.216)]^{-1} = 0.82$$
- $$\mathbb {P}(Obs \mid \varphi _2) = [1 + (0.343)]^{-1} = 0.74$$

After normalizing the probabilities using the normalization factor $\eta$^2^, and assuming that the prior probability $\mathbb {P}(\varphi )$ is equal to every goal in the set of goals $\mathcal {G}_{\varphi }$, (6) computes the posterior probabilities (1) for the temporally extended goals $\mathcal {G}_{\varphi }$. A *solution* to a recognition problem $\mathcal {T}_{\varphi }$ (Definition 8) is a set of temporally extended goals $\mathcal {G}_{\varphi }^{*}$ with the *maximum probability*: $\mathcal {G}_{\varphi }^{*} = \mathop {\mathrm {arg\,max}}\limits _{\varphi \in \mathcal {G}_{\varphi }} \mathbb {P}(\varphi \mid Obs)$. Hence, considering the normalizing factor $\eta$ and the probabilities $\mathbb {P}(Obs \mid \varphi )$ computed before, we then have the following posterior probabilities for the goals in Example 2: $\mathbb {P}(\varphi _0 \mid Obs) = 0.001$; $\mathbb {P}(\varphi _1 \mid Obs) = 0.524$; and $\mathbb {P}(\varphi _2 \mid Obs) = 0.475$. Recall that in Example 2, $\varphi ^{*}$ is $\varphi _1$, and according to the computed posterior probabilities, we then have $\mathcal {G}_{\varphi }^{*} = \lbrace \varphi _1 \rbrace$, so our approach yields only the intended goal by observing just two observations.

Using the *average distance* $\textbf{d}$ and the *penalty value* $\textbf{p}$ allows our approach to disambiguate similar goals during the recognition stage. For instance, consider the following possible temporally extended goals: $\varphi _0 = \phi _1 \mathcal {U} \phi _2$ and $\varphi _1 = \phi _2 \mathcal {U} \phi _1$. Here, both goals have the same formulas to be achieved, i.e., $\phi _1$ and $\phi _2$, but in a different order. Thus, even having the same formulas to be achieved, the sequences of their policies’ executions are different. Therefore, the average distances are also different, possibly a smaller value for the temporally extended goal that the agent aims to achieve, and the penalty value may also be applied to the other goal if two subsequent observations do not have any order relation in the set of executions for this goal.

#### Computational analysis

The most expensive computational part of our recognition approach is computing the policies $\pi$ for the goal hypotheses $\mathcal {G}_{\varphi }$. Thus, our approach requires $|\mathcal {G}_{\varphi }|$ calls to an off-the-shelf fond planner. Hence, the computational complexity of our recognition approach is linear in the number of goal hypotheses $|\mathcal {G}_{\varphi }|$. In contrast, to recognize goals and plans in *Classical Planning* settings, the approach of [35] requires $2 * |\mathcal {G}|$ calls to an off-the-shelf *Classical* planner. Concretely, to compute $\mathbb {P}(Obs \mid G)$, Ramirez and Geffner’s approach computes two plans for every goal and based on these two plans, they compute a *cost-difference* between these plans and plug it into a Boltzmann equation. For computing these two plans, this approach requires a non-trivial transformation process that modifies both the domain and problem, i.e., an augmented domain and problem that compute a plan that *complies* with the observations, and another augmented domain and problem to compute a plan that *does not comply* with the observations. Essentially, the intuition of Ramirez and Geffner’s approach is that the lower the *cost-difference* for a goal, the higher the probability for this goal, much similar to the intuition of our *estimated score* $\mathcal {E}$.

## Experiments and evaluation

This section details experiments and evaluations carried out to validate the effectiveness of our recognition approach. The empirical evaluation covers thousands of goal recognition problems using well-known fond planning domain models with different types of temporally extended goals expressed in ltl$_f$ and ppltl.

The source code of our PDDL encoding for ltl$_f$ and ppltl goals^3^ and our temporally extended goal recognition approach^4^, as well as the recognition datasets and results are available on GitHub.

### Domains, recognition datasets, and setup

The experiments and evaluation analysis employ six different well-known fond planning domain models: Blocks-World, Logistics, Tidy-up, Tireworld, Triangle-Tireworld, and Zeno-Travel. Most of them are commonly used in the AI Planning community to evaluate fond planners [23, 25]. The domain models involve practical real-world applications, such as navigating, stacking, picking up and putting down objects, loading and unloading objects, loading and unloading objects, and etc. Some domains combine more than one of the characteristics above, namely, Logistics, Tidy-up [26], and Zeno-Travel, which involve navigating and manipulating objects in the environment. In practice, our recognition approach is capable of recognizing not only the set of facts of a goal that an observed agent aims to achieve from a sequence of observations, but also the *temporal order* (e.g., *exact order*) in which the agent aims to achieve this set of facts that represents a temporally extended goal. For instance, for Tidy-up, is a real-world application domain, in which the purpose is defining planning tasks for a household robot that could assist elder people in smart-home application, our approach would be able to monitor and assist the household robot to achieve its goals in a specific order.

Based on these fond planning domain models, we build different recognition datasets: a *baseline* dataset using conjunctive goals ($\phi _1\wedge \phi _2$) and datasets with ltl$_f$ and ppltl goals.

The ltl$_f$ datasets use three types of goals:

- $\Diamond \phi$, where $\phi$ is a propositional formula expressing that *eventually* $\phi$ will be achieved. This temporal formula is analogous to a reachability goal;
- $\Diamond (\phi _1 \wedge {{\circ }{} }(\Diamond \phi _2))$, expressing that $\phi _1$ must hold before $\phi _2$ holds. For instance, we can define a temporal goal that expresses the order in which a set of packages in Logistics domain should be delivered;
- $\phi _1 \mathcal {U} \phi _2$: $\phi _1$ must hold *until* $\phi _2$ is achieved. For the Tidy-up domain, we can define a temporal goal that no one can be in the kitchen until the robot cleans the kitchen.

The ppltl datasets use two types of goals:

, expressing that $\phi _1$ holds and $\phi _2$ held once. For instance, in the Blocks-World domain, we can define a past temporal goal that only allows stacking a set of blocks (a, b, c) once another set of blocks has been stacked (d, e);
- $\phi _1 \wedge (\lnot \phi _2 \mathcal {S} \phi _3)$, expressing that the formula $\phi _1$ holds and *since* $\phi _3$ held $\phi _2$ was not true anymore. For instance, in Zeno-Travel, we can define a past temporal goal expressing that person1 is at city1 and since the person2 is at city1, the aircraft must not pass through city2 anymore.

Thus, in total, there are six different recognition datasets over the six fond planning domains and temporal formulas presented above. Each of these datasets contains hundreds of recognition problems ($\approx$ 390 recognition problems per dataset), such that each recognition problem $\mathcal {T}_{\varphi }$ in these datasets is comprised of a fond planning domain model $\mathcal {D}$, an initial state $s_0$, a set of possible goals $\mathcal {G}_{\varphi }$ (expressed in either ltl$_f$ or ppltl), the actual intended hidden goal in the set of possible goals $\varphi ^{*} \in \mathcal {G}_{\varphi }$, and the observation sequence *Obs*. Note that the set of possible goals $\mathcal {G}_{\varphi }$ contains very similar goals (i.e., $\varphi _0 = \phi _1 \mathcal {U} \phi _2$ and $\varphi _1 = \phi _2 \mathcal {U} \phi _1$), and all possible goals can be achieved from the initial state by a strong-cyclic policy. For instance, for the Tidy-up domain, we define the following ltl$_f$ goals as possible goals $\mathcal {G}_{\varphi }$:

- $$\varphi _0 = \Diamond (\texttt {(wiped desk1)} \wedge {{\circ }{} }(\Diamond \texttt {(on book1 desk1)}))$$;
- $$\varphi _1 = \Diamond (\texttt {(on book1 desk1)} \wedge {{\circ }{} }(\Diamond \texttt {(wiped desk1)}))$$;
- $$\varphi _2 = \Diamond (\texttt {(on cup1 desk2)} \wedge {{\circ }{} }(\Diamond \texttt {(wiped desk2)}))$$;
- $$\varphi _3 = \Diamond (\texttt {(wiped desk2)} \wedge {{\circ }{} }(\Diamond \texttt {(on cup1 desk2)}))$$;

Note that some of the goals described above share the same formulas and fluents, but some of these formulas must be achieved in a different order, e.g., $\varphi _0$ and $\varphi _1$, and $\varphi _2$ and $\varphi _3$. Note that our recognition approach is very accurate in discerning (Table 1) the order that the intended goal aims to be achieved based on few observations (executions of the agent in the environment).

As mentioned earlier in the paper, an observation sequence contains a sequence of actions that represent an execution $\vec {e}$ in the set of possible executions $\vec {E}$ of policy $\pi$ that achieves the actual intended hidden goal $\varphi ^{*}$, and as before, this observation sequence *Obs* can be full or partial. To generate the observations *Obs* for $\varphi ^{*}$ and build the recognition problems, our approach extracts strong-cyclic policies using different fond planners, such as PRP and MyND. A full observation sequence represents an execution (a sequence of executed actions) of a strong-cyclic policy that achieves the actual intended hidden goal $\varphi ^{*}$, i.e., 100% of the actions of $\vec {e}$ being observed. A partial observation sequence is represented by a sub-sequence of actions of a full execution that aims to achieve the actual intended hidden goal $\varphi ^{*}$ (e.g., an execution with “missing” actions, due to a sensor malfunction). In our recognition datasets, we define four levels of observability for a partial observation sequence: 10%, 30%, 50%, or 70% of its actions being observed. For instance, for a full observation sequence *Obs* with 10 actions (100% of observability), a corresponding partial observations sequence with 10% of observability would have only one observed action, and for 30% of observability three observed actions, and so on for the other levels of observability.

We ran all experiments using PRP [25] planner with a single core of a 12 core Intel(R) Xeon(R) CPU E5-2620 v3 @ 2.40GHz with 16GB of RAM, set a maximum memory usage limit of 8GB, and set a 10-minute timeout for each recognition problem. We are unable to provide a *direct comparison* of our approach against existing recognition approaches in the literature because most of these approaches perform a non-trivial process that transforms a recognition problem into planning problems to be solved by a planner [35, 37]. Even adapting such a transformation to work in fond settings with temporally extended goals, one cannot guarantee that it will work properly in the problem setting introduced in this paper.

**Table 1:** Offline Recognition results for Conjunctive, ltl$_f$, and ppltl goals

	$|\mathcal {G}_{\phi }|$	|*Obs*|	*Time*	*TPR*	*FPR*	*FNR*	*F1-Score*	*Time*	*TPR*	*FPR*	*FNR*	*F1-Score*
			Conjunctive Goals					ltl$_f$ Eventuality Goals				
			$\phi _1 \wedge \phi _2$					$\Diamond \phi$				
10	5.2	3.85	189.1	0.75	0.15	0.25	0.63	243.8	0.74	0.11	0.26	0.60
30		10.7	187.2	0.85	0.08	0.15	0.78	235.1	0.86	0.10	0.14	0.78
50		17.4	188.4	0.83	0.09	0.17	0.82	242.1	0.89	0.07	0.11	0.92
70		24.3	187.8	0.86	0.08	0.14	0.84	232.1	0.92	0.08	0.08	0.87
100		34.7	190.4	0.85	0.09	0.15	0.86	272.8	0.95	0.09	0.05	0.90
			ltl$_f$ Ordering Goals					ltl$_f$ Goals Until				
			$$\Diamond (\phi _1 \wedge $$ $${\circ }$$$$(\Diamond \phi _2))$$					$$\phi _1 \mathcal {U} \phi _2$$				
10	4.0	2.1	136.1	0.68	0.15	0.32	0.62	217.9	0.79	0.11	0.21	0.72
30		5.4	130.9	0.84	0.13	0.16	0.76	215.8	0.91	0.12	0.09	0.82
50		8.8	132.1	0.88	0.10	0.12	0.80	210.1	0.93	0.10	0.07	0.83
70		12.5	129.2	0.95	0.06	0.05	0.89	211.5	0.97	0.09	0.03	0.86
100		17.1	126.6	0.94	0.05	0.06	0.90	207.7	0.97	0.07	0.03	0.87
			ppltl Goals Once					ppltl Goals Since				
								$$\phi _1 \wedge (\lnot \phi _2 \mathcal {S} \phi _3)$$				
10	4.0	1.7	144.8	0.73	0.11	0.27	0.67	173.5	0.76	0.18	0.24	0.64
30		4.6	141.3	0.84	0.07	0.16	0.79	173.3	0.87	0.12	0.13	0.78
50		7.3	141.9	0.89	0.08	0.11	0.82	172.9	0.85	0.09	0.15	0.79
70		10.3	142.9	0.95	0.07	0.05	0.87	171.1	0.97	0.07	0.03	0.91
100		14.2	155.8	0.97	0.07	0.03	0.88	169.3	0.94	0.02	0.06	0.93

### Evaluation metrics

Our evaluation uses widely known metrics in the *Goal and Plan Recognition* literature [31, 34, 39]. To evaluate our approach in the *Offline Keyhole Recognition* setting, we use four metrics, as follows:

- *True Positive Rate* (*TPR*) measures the fraction of times that the intended hidden goal $\varphi ^{*}$ was correctly recognized, e.g., the percentage of recognition problems that our approach correctly recognized the intended goal. A **higher** *TPR* indicates better accuracy, measuring how often the intended hidden goal had the highest probability $P(\varphi \mid Obs)$ among the possible goals. *TPR* (7) is the ratio between true positive results^5^, and the sum of true positive and false negative results^6^;
  7 $$\begin{aligned} TPR = \frac{TP}{TP + FN} = 1 - FNR \end{aligned}$$
- *False Positive Rate* (*FPR*) is a metric that measures how often goals other than the intended goal are recognized (wrongly) as the intended ones. A **lower** *FPR* indicates better accuracy. *FPR* is the ratio between false positive results^7^, and the sum of false positive and true negative results^8^;
  8 $$\begin{aligned} FPR = \frac{FP}{FP + TN} \end{aligned}$$
- *False Negative Rate* (*FNR*) aims to measure the fraction of times in which the intended correct goal was recognized incorrectly. A **lower** *FNR* indicates better accuracy. *FNR* (9) is the ratio between false negative results and the sum of false negative and true positive results;
  9 $$\begin{aligned} FNR = \frac{FN}{FN + TP} = 1 - TPR \end{aligned}$$
- *F1-Score* (10) is the harmonic mean of precision and sensitivity (i.e., *TPR*), representing the trade-off between true positive and false positive results. The **highest possible value** of an *F1-Score* is 1.0, indicating perfect precision and sensitivity, and the **lowest possible value** is 0. Thus, **higher** *F1-Score* values indicate better accuracy.
  10 $$\begin{aligned} F1-Score = \frac{2*TP}{2TP + FP + FN} \end{aligned}$$

In contrast, to evaluate our approach in the *Online Recognition* setting, we use the following metric:

- *Ranked First* is a metric that measures the number of times the intended goal hypothesis $\varphi ^{*}$ has been correctly ranked first as the most likely intended goal, and **higher** values for this metric indicate better accuracy for performing online recognition.

**Fig. 5:**
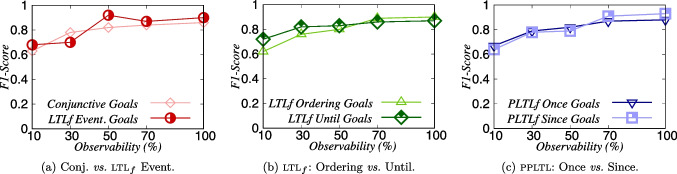
F1-Score comparison

In addition to the metrics mentioned above, we also evaluate our recognition approach in terms of *recognition time* (*Time*), which is the average time in seconds to perform the recognition process (including the calls to a fond planner);

### Offline keyhole recognition results

We now assess how accurate our recognition approach is in the *Keyhole Recognition* setting. Table 1 shows three inner tables that summarize and aggregate the average results of all the six datasets for four different metrics, such as *Time*, *TPR*, *FPR*, and *FNR*. $|\mathcal {G}_{\varphi }|$ represents the average number of goals in the datasets, and |*Obs*| the average number of observations. Each row in these inner tables represents the observation level, varying from 10% to 100%. Figure 5 shows the performance of our approach by comparing the results using *F1-Score* for the six types of temporal formulas we used for evaluation. Table 2 shows in much more detail the results for each of the six datasets that have been used for evaluating of our recognition approach.

#### Offline results for conjunctive and eventuality goals

The first inner table shows the average results comparing the performance of our approach between conjunctive goals and temporally extended goals using the *eventually* temporal operator $\Diamond$. We refer to this comparison as the *baseline* since these two types of goals have the same semantics. We can see that the results for these two types of goals are very similar for all metrics. Moreover, it is also possible to see that our recognition approach is very accurate and performs well at all levels of observability, yielding high *TPR* values and low *FPR* and *FNR* values for more than 10% of observability. Note that for 10% of observability, and ltl$_f$ goals for $\Diamond \varphi$, the *TPR* average value is 0.74, and it means for 74% of the recognition problems our approach recognized correctly the intended temporally extended goal when observing, on average, only 3.85 actions. Figure 5a shows that our approach yields higher *F1-Score* values (i.e., greater than 0.79) for these types of formulas when dealing with more than 50% of observability.

#### Offline results for ltl$_f$ goals

Regarding the results for the two types of ltl$_f$ goals (second inner table), it is possible to see that our approach shows to be accurate for all metrics at all levels of observability, apart from the results for 10% of observability for ltl$_f$ goals in which the formulas must be recognized in a certain order. Note that our approach is accurate even when observing just a few actions (2.1 for 10% and 5.4 for 30%), but not as accurate as for more than 30% of observability. Figure 5b shows that our approach yields higher *F1-Score* values (i.e., greater than 0.75) when dealing with more than 30% of observability.

#### Offline results for ppltl goals

Finally, as for the results for the two types of ppltl goals, it is possible to observe in the last inner table that the overall average number of observations |*Obs*| is less than the average for the other datasets, making the task of goal recognition more difficult for the ppltl datasets. Yet, our recognition approach remains accurate when dealing with fewer observations. Moreover, the values of *FNR* increase for low observability, but the *FPR* values are, on average, inferior to $\approx$ 0.15. Figure 5c shows that our approach gradually increases the *F1-Score* values when also increases the percentage of observability.

**Table 2:** Offline Recognition experimental results for all six fond domains separately

	Conjunctive Goals						ltl$_f$ Eventuality Goals						ltl$_f$ Ordering					
	$$\phi _1 \wedge \phi _2$$						$$\Diamond \phi $$						$$\Diamond (\phi _1 \wedge $$ $${\circ }$$$$(\Diamond \phi _2))$$					
	$|\mathcal {G}_{\varphi }|$	|*Obs*|	*Time*	*TPR*	*FPR*	*FNR*	$|\mathcal {G}_{\varphi }|$	|*Obs*|	*Time*	*TPR*	*FPR*	*FNR*	$|\mathcal {G}_{\varphi }|$	|*Obs*|	*Time*	*TPR*	*FPR*	*FNR*
Blocks-World																		
10	6.0	3.92	33.53	0.81	0.10	0.19	6.0	3.92	81.16	0.81	0.06	0.19	4.0	1.33	38.05	0.67	0.10	0.33
30		10.33	33.91	0.86	0.09	0.14		10.58	69.65	0.81	0.10	0.19		3.17	39.49	0.83	0.07	0.17
50		16.67	33.90	0.75	0.14	0.25		17.08	67.16	0.72	0.09	0.28		4.67	39.63	0.89	0.07	0.11
70		23.58	33.98	0.75	0.13	0.25		23.92	67.71	0.78	0.09	0.22		6.67	38.92	0.89	0.07	0.11
100		33.00	34.01	0.75	0.17	0.25		33.58	68.82	0.75	0.14	0.25		9.00	38.28	0.83	0.08	0.17
Logistics																		
10	4.0	3.00	260.92	0.85	0.11	0.15	4.0	3.00	412.34	0.70	0.11	0.30	4.0	2.33	498.70	0.72	0.22	0.28
30		8.11	258.60	0.89	0.08	0.11		8.11	360.49	0.96	0.10	0.04		5.83	468.36	0.89	0.17	0.11
50		13.11	258.58	0.89	0.09	0.11		13.00	383.45	1.00	0.09	0.00		9.17	480.20	0.89	0.19	0.11
70		18.33	251.51	0.96	0.09	0.04		18.11	380.51	1.00	0.09	0.00		13.00	466.86	1.00	0.11	0.00
100		25.44	251.27	1.00	0.08	0.00		25.22	444.65	1.00	0.08	0.00		17.83	450.51	1.00	0.08	0.00
Tidyup																		
10	4.0	6.56	180.17	0.37	0.27	0.63	4.0	7.00	230.36	0.52	0.20	0.48	4.0	3.50	106.87	0.67	0.28	0.33
30		18.78	178.05	0.48	0.19	0.52		20.00	228.34	0.63	0.32	0.37		9.50	105.93	0.67	0.36	0.33
50		31.00	179.45	0.44	0.22	0.56		32.89	191.61	0.81	0.22	0.19		15.50	105.84	0.78	0.28	0.22
70		43.56	178.79	0.41	0.22	0.59		46.11	193.81	0.81	0.31	0.19		21.83	104.01	0.89	0.19	0.11
100		61.56	179.52	0.33	0.25	0.67		65.33	247.09	1.00	0.28	0.00		30.50	100.16	0.83	0.12	0.17
Tireworld																		
10	5.5	1.50	16.88	1.00	0.29	0.00	5.5	1.50	29.17	1.00	0.19	0.00	3.5	1.50	12.59	0.67	0.16	0.33
30		3.50	17.19	1.00	0.04	0.00		3.50	26.39	1.00	0.07	0.00		3.50	11.90	0.72	0.16	0.28
50		6.00	17.35	1.00	0.01	0.00		6.00	21.97	1.00	0.04	0.00		5.67	10.18	0.89	0.05	0.11
70		8.50	17.31	1.00	0.01	0.00		8.50	20.30	1.00	0.00	0.00		7.83	10.20	0.94	0.02	0.06
100		11.50	17.28	1.00	0.00	0.00		11.50	20.97	1.00	0.00	0.00		10.50	10.15	1.00	0.00	0.00
Triangle-Tireworld																		
10	3.75	1.67	16.56	0.64	0.16	0.36	3.75	2.08	34.57	0.69	0.13	0.31	4.0	1.67	14.42	0.44	0.14	0.56
30		4.67	16.90	0.86	0.03	0.14		5.58	31.76	0.86	0.03	0.14		3.83	14.57	0.94	0.01	0.06
50		7.33	17.08	0.89	0.03	0.11		8.83	30.83	0.92	0.02	0.08		6.17	14.76	0.83	0.04	0.17
70		10.00	17.12	1.00	0.00	0.00		12.08	32.81	1.00	0.00	0.00		8.50	17.43	1.00	0.00	0.00
100		13.67	17.16	1.00	0.00	0.00		16.67	30.93	1.00	0.00	0.00		11.33	24.17	1.00	0.00	0.00
Zeno-Travel																		
10	7.5	5.67	556.36	0.89	0.08	0.11	7.5	5.33	607.20	0.81	0.05	0.19	4.0	2.67	145.81	0.94	0.01	0.06
30		16.25	549.34	1.00	0.04	0.00		15.17	619.13	0.94	0.02	0.06		7.50	145.33	1.00	0.00	0.00
50		26.50	554.09	1.00	0.02	0.00		24.75	670.07	0.97	0.02	0.03		11.67	141.81	1.00	0.00	0.00
70		37.50	556.76	1.00	0.03	0.00		34.92	619.24	1.00	0.02	0.00		16.67	138.04	1.00	0.00	0.00
100		53.00	569.43	1.00	0.02	0.00		49.42	735.20	1.00	0.02	0.00		23.17	136.37	1.00	0.00	0.00

	ltl$_f$ Goals Until						ppltl Goals Once						ppltl Goals Since					
	$\phi _1 \mathcal {U} \phi _2$												$\phi _1 \wedge (\lnot \phi _2 \mathcal {S} \phi _3)$					
	$|\mathcal {G}_{\varphi }|$	|*Obs*|	*Time*	*TPR*	*FPR*	*FNR*	$|\mathcal {G}_{\varphi }|$	|*Obs*|	*Time*	*TPR*	*FPR*	*FNR*	$|\mathcal {G}_{\varphi }|$	|*Obs*|	*Time*	*TPR*	*FPR*	*FNR*
Blocks-World																		
10	4.0	1.00	342.01	0.72	0.08	0.28	4.0	1.17	38.97	0.89	0.08	0.11	4.0	1.00	50.98	0.89	0.14	0.11
30		2.83	357.48	0.94	0.01	0.06		3.17	39.15	1.00	0.03	0.00		2.83	53.42	1.00	0.00	0.00
50		3.83	349.43	1.00	0.00	0.00		4.67	38.32	1.00	0.06	0.00		3.83	50.67	1.00	0.00	0.00
70		5.83	355.37	1.00	0.00	0.00		6.67	38.16	1.00	0.07	0.00		5.83	48.33	1.00	0.00	0.00
100		7.67	393.83	1.00	0.00	0.00		9.17	37.92	1.00	0.08	0.00		7.67	47.21	1.00	0.00	0.00
Logistics																		
10	4.0	1.83	310.16	1.00	0.12	0.00	4.0	1.67	554.45	0.78	0.14	0.22	4.0	1.00	643.01	0.83	0.14	0.17
30		4.67	292.87	0.94	0.32	0.06		4.17	541.31	0.94	0.12	0.06		2.33	645.55	0.83	0.18	0.17
50		7.67	282.77	1.00	0.31	0.00		6.33	542.76	0.94	0.12	0.06		3.17	652.51	0.89	0.15	0.11
70		11.00	285.97	1.00	0.28	0.00		9.00	552.88	1.00	0.08	0.00		4.50	648.61	1.00	0.05	0.00
100		14.83	232.00	1.00	0.17	0.00		12.50	630.17	1.00	0.08	0.00		6.00	644.07	1.00	0.00	0.00
Tidyup																		
10	4.0	3.17	45.20	0.72	0.22	0.28	4.0	3.33	108.24	0.67	0.11	0.33	4.0	3.50	47.25	0.50	0.31	0.50
30		8.33	45.53	0.78	0.26	0.22		9.00	104.46	0.61	0.14	0.39		10.33	46.47	0.56	0.21	0.44
50		13.50	43.40	0.89	0.25	0.11		14.50	105.02	0.72	0.17	0.28		17.00	45.71	0.33	0.24	0.67
70		19.33	43.17	0.94	0.24	0.06		20.33	106.25	0.89	0.19	0.11		23.67	48.27	0.83	0.08	0.17
100		26.83	43.97	1.00	0.17	0.00		28.50	107.56	1.00	0.21	0.00		33.50	48.22	0.67	0.08	0.33
Tireworld																		
10	3.5	1.17	5.11	0.72	0.08	0.28	4.0	1.33	6.85	0.56	0.19	0.44	4.0	1.17	18.00	0.67	0.12	0.33
30		3.17	5.17	0.94	0.03	0.06		3.50	6.95	0.83	0.10	0.17		3.17	18.69	0.89	0.07	0.11
50		4.83	5.10	0.94	0.01	0.06		5.50	6.87	0.83	0.10	0.17		4.83	19.09	0.94	0.04	0.06
70		6.50	5.16	1.00	0.06	0.00		7.67	6.82	0.83	0.07	0.17		6.50	19.23	1.00	0.04	0.00
100		9.00	5.55	1.00	0.08	0.00		9.00	5.55	1.00	0.08	0.00		9.00	19.17	1.00	0.04	0.00
Triangle-Tireworld																		
10	4.0	1.17	11.04	0.72	0.11	0.28	4.0	1.67	15.91	0.67	0.11	0.33	4.0	1.00	106.56	0.78	0.26	0.22
30		2.83	10.55	1.00	0.03	0.00		3.83	15.87	0.72	0.07	0.28		2.33	104.83	0.94	0.17	0.06
50		4.50	10.55	0.94	0.01	0.06		6.17	16.13	0.89	0.03	0.11		3.50	102.41	1.00	0.12	0.00
70		6.17	10.58	1.00	0.00	0.00		8.50	14.87	1.00	0.00	0.00		4.67	102.36	1.00	0.25	0.00
100		8.00	11.89	1.00	0.00	0.00		11.33	15.16	1.00	0.00	0.00		6.00	102.91	1.00	0.00	0.00
Zeno-Travel																		
10	4.0	2.50	174.38	0.89	0.03	0.11	4.0	2.17	144.87	0.83	0.04	0.17	4.0	2.00	175.58	0.89	0.14	0.11
30		6.50	167.33	0.89	0.03	0.11		6.17	140.15	0.94	0.01	0.06		5.33	171.27	1.00	0.10	0.00
50		10.17	164.68	0.83	0.04	0.17		9.83	142.72	0.94	0.01	0.06		8.67	167.39	0.94	0.04	0.06
70		14.33	161.67	0.89	0.03	0.11		13.83	138.64	1.00	0.00	0.00		12.67	159.45	1.00	0.00	0.00
100		20.00	161.05	0.83	0.04	0.17		19.50	137.53	1.00	0.00	0.00		17.33	154.69	1.00	0.00	0.00

### Online recognition results

With the experiments and evaluation in the *Keyhole Offline* recognition setting in place, we now proceed to present the experiments and evaluation in the *Online* recognition setting. As before, performing the recognition task in the *Online* recognition setting is usually harder than in the offline setting, as the recognition task has to be performed incrementally and gradually, and the recognizer sees the observations step-by-step, rather than performing the recognition task by analyzing all observations at once, as in the offline recognition setting.

**Fig. 6:**
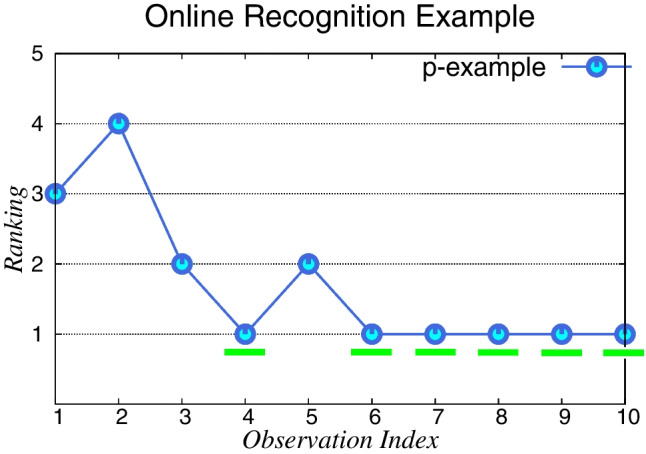
Online Recognition example

Figure 6 exemplifies the evaluation in the *Online* recognition setting. This uses the *Ranked First* metric, which measures how many times over the observation sequence the correct intended goal $\varphi ^{*}$ has been ranked first as the *top-1* goal over the goal hypotheses $\mathcal {G}_{\varphi }$. The recognition problem example depicted in Fig. 6 has five goal hypotheses (y-axis), and ten actions in the observation sequence (x-axis). As stated before, the recognition task in the *Online* setting is done gradually, step-by-step, so at every step our approach essentially ranks the goals according to the probability distribution over the goal hypotheses $\mathcal {G}_{\varphi }$. The example in Fig. 6 shows the correct goal $\varphi ^{*}$ *Ranked First* six times (at the observation indexes: 4, 6, 7, 8, 9, and 10) over the observation sequence with ten observation, so it means that the goal correct intended goal $\mathcal {G}_{\varphi }$ is *Ranked First* (i.e., as the *top-1*, with the highest probability among the goal hypotheses $\mathcal {G}_{\varphi }$) 60% of the time in the observation sequence for this recognition example.

**Fig. 7:**
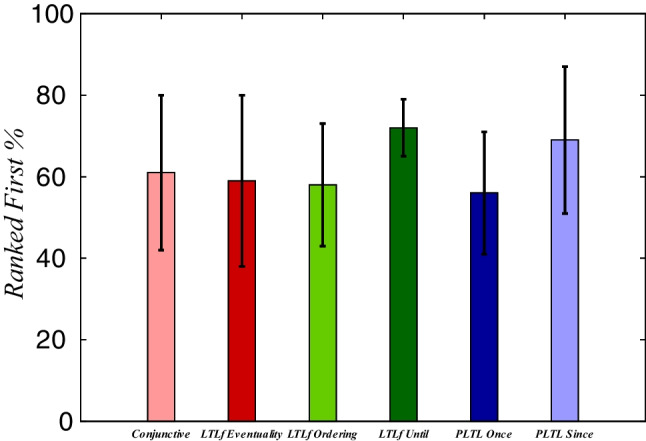
Online Recognition Histogram

Figure 7 aggregates the average recognition results of all the six datasets for the *Ranked First* metric as a histogram, by considering full observation sequences that represent executions (sequences of executed actions) of strong-cyclic policies that achieves the actual intended goal $\varphi ^{*}$. The results represent the overall percentage (including the standard deviation – black bars) of how many times the of time that the correct intended goal $\varphi ^{*}$ has been ranked first over the observations. The average results indicated our approach to be in general accurate to recognize correctly the *temporal order* of the facts in the goals in the *Online* recognition setting, yielding *Ranked First* percentage values greater than 58%.

**Fig. 8:**
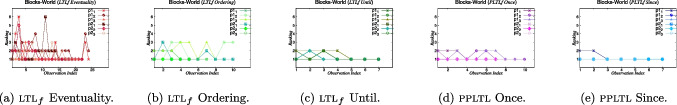
Online recognition ranking over the observations for Blocks-World

**Fig. 9:**
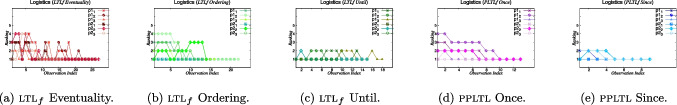
Online recognition ranking over the observations for Logistics

**Fig. 10:**
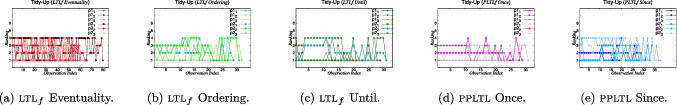
Online recognition ranking over the observations for Tidy-Up

**Fig. 11:**
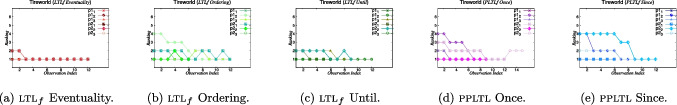
Online recognition ranking over the observations for Tireworld

**Fig. 12:**
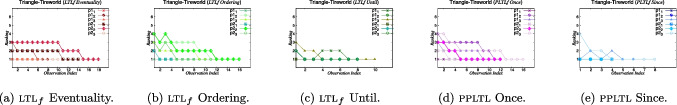
Online recognition ranking over the observations for Triangle-Tireworld

**Fig. 13:**
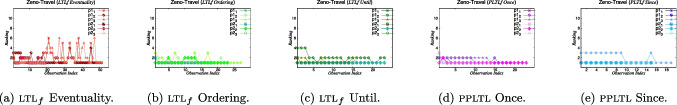
Online Recognition ranking over the observations for Zeno-Travel

Figures 8, 9, 10, 11, 10, 12, and 13 shows the *Online* recognition results separately for all six domains models and the different types of temporally extended goals. By analyzing the *Online* recognition results more closely, one can see that our approach converges to rank the correct goal as the *top-1* mostly after a few observations. This means that it is commonly hard to disambiguate among the goals at the beginning of the execution, which, in turn, directly affects the overall *Ranked First* percentage values (as shown in Fig. 7). Here, our approach struggles to disambiguate and recognize correctly the intended goal for some recognition problems and some types of temporal formulas. Namely, our approach has struggled to disambiguate when dealing with ltl$_f$ Eventuality goals in Blocks-World (see Fig. 8a), for most temporal extended goals in Tidy-Up (see Fig. 10), and for ltl$_f$ Eventuality goals in Zeno-Travel (see Fig. 13a).

## Related work and discussion

To the best of our knowledge, existing approaches to *Goal and Plan Recognition as Planning* cannot explicitly recognize temporally extended goals in non-deterministic environments. Seminal and recent work on *Goal Recognition as Planning* relies on deterministic planning techniques [31, 34, 37] for recognizing conjunctive goals. By contrast, we propose a novel problem formalization for goal recognition, addressing temporally extended goals (ltl$_f$ or ppltl goals) in fond planning domain models. While our probabilistic approach relies on the probabilistic framework of [35], we address the challenge of computing $\mathbb {P}(Obs \mid G)$ in a completely different way.

There exist different techniques to *Goal and Plan Recognition* in the literature, including approaches that rely on plan libraries [4], context-free grammars [19], and Hierarchical Task Network (HTN) [21]. Such approaches rely on hierarchical structures that represent the knowledge of how to achieve the possible goals, and this knowledge can be seen as potential strategies for achieving the set of possible goals. Note that the temporal constraints of temporally extended goals can be adapted and translated to such hierarchical knowledge. For instance, context-free grammars are expressive enough to encode temporally extended goals [11]. ltl$_f$ has the expressive power of the star-free fragment of regular expressions and hence captured by context-free grammars. However, unlike regular expressions, ltl$_f$ uses negation and conjunction liberally, and the translation to regular expression is computationally costly. Note, being equally expressive is not a meaningful indication of the complexity of transforming one formalism into another. [17] show that, while ltl$_f$ and ppltl have the same expressive power, the best translation techniques known are worst-case 3EXPTIME.

As far as we know, there are no encodings of ltl$_f$-like specification languages into HTN, and its difficulty is unclear. Nevertheless, combining HTN and ltl$_f$ could be interesting for further study. HTN techniques focus on the knowledge about the decomposition property of traces, whereas ltl$_f$-like solutions focus on the knowledge about dynamic properties of traces, similar to what is done in verification settings. Most recently, [7] develop a novel Pure-Past Linear Temporal Logic PDDL encoding for planning in the *Classical Planning* setting.

## Conclusions

This article introduced a novel problem formalization for recognizing *temporally extended goals*, specified in either ltl$_f$ or ppltl, in fond planning domain models. It also developed a novel probabilistic framework for goal recognition in such settings, and implemented a compilation of temporally extended goals that allows us to reduce the problem of fond planning for ltl$_f$/ppltl goals to standard fond planning. Our experiments have shown that our recognition approach yields high accuracy for recognizing temporally extended goals (ltl$_f$/ppltl) in different settings (*Keyhole Offline* and *Online* recognition) at several levels of observability.

As future work, we intend to extend and adapt our recognition approach for being able to deal with spurious (noisy) observations, and recognize not only the temporal extended goals but also anticipate the policy that the agent is executing to achieve its goals.

## Footnotes

1 The fairness assumption defines that all action outcomes in a given state have a non-zero probability.

2 $$\eta = [\sum _{\varphi \in \mathcal {G}_{\varphi }} \mathbb {P}(Obs \mid \varphi ) * \mathbb {P}(\varphi )]^{-1}$$

3 https://github.com/whitemech/FOND4LTLf

4 https://github.com/ramonpereira/goal-recognition-ltlf_pltlf-fond

5 *True positive results* represent the number of goals that has been recognized correctly.

6 *False negative results* represent the number of correct goals that has not been recognized.

7 *False positive results* are the number of incorrect goals that has been recognized as the correct ones.

8 *True negative results* represent the number of incorrect goals has been recognized correctly as the incorrect ones.
